# Clothing insulation and temperature, layer and mass of clothing under comfortable environmental conditions

**DOI:** 10.1186/1880-6805-32-11

**Published:** 2013-07-01

**Authors:** JuYoun Kwon, Jeongwha Choi

**Affiliations:** 1 Division for Design & Human Engineering, UNIST Multidisciplinary Institute, Ulsan National Institute of Science and Technology, Ulsan, South Korea; 2 Department of Clothing and Textiles/Research Institute of Human Ecology, College of Human Ecology, Seoul National University, Seoul, South Korea

## Abstract

This study was designed to investigate the relationship between the microclimate temperature and clothing insulation (*I*_*cl*_) under comfortable environmental conditions. In total, 20 subjects (13 women, 7 men) took part in this study. Four environmental temperatures were chosen: 14°C (to represent March/April), 25°C (May/June), 29°C (July/August), and 23°C (September/October). Wind speed (0.14ms^-1^) and humidity (45%) were held constant. Clothing microclimate temperatures were measured at the chest (*T*_*chest*_) and on the interscapular region (*T*_*scapular*_). Clothing temperature of the innermost layer (*T*_*innermost*_) was measured on this layer 30 mm above the centre of the left breast. Subjects were free to choose the clothing that offered them thermal comfort under each environmental condition. We found the following results. 1) All clothing factors except the number of lower clothing layers (*L*_*lower*_), showed differences between the different environmental conditions (P<0.05). The ranges of *T*_*chest*_ were 31.6 to 33.5°C and 32.2 to 33.4°C in *T*_*scapular*_. The range of *T*_*innermost*_ was 28.6 to 32.0°C. The range of the upper clothing layers (*L*_*upper*_) and total clothing mass (*M*_*total*_) was 1.1 to 3.2 layers and 473 to 1659 g respectively. The range of *I*_*cl*_ was 0.78 to 2.10 clo. 2) *Post hoc* analyses showed that analysis of *T*_*innermost*_ produced the same results as for that of *I*_*cl*_. Likewise, the analysis of *L*_*upper*_ produced the same result as the analysis of the number of total layers (*L*_*total*_) within an outfit. 3) Air temperature (*t*_*a*_) had positive relationships with *T*_*chest*_ and *T*_*scapular*_ and with *T*_*innermost*_ but had inverse correlations with *I*_*cl*_, *M*_*total*_, *L*_*upper*_ and *L*_*total*_. *T*_*chest*_, *T*_*scapular*_, and *T*_*innermost*_ increased as *t*_*a*_ rose. 4) *I*_*cl*_ had inverse relationships with *T*_*chest*_ and *T*_*innermost*_, but positive relationships with *M*_*total*_, *L*_*upper*_ and *L*_*total*_. *I*_*cl*_ could be estimated by *M*_*total*_, *L*_*upper*_, and *T*_*scapular*_ using a multivariate linear regression model. 5) *L*_*upper*_ had positive relationships with *I*_*cl*_ and *M*_*total*_, but *L*_*lower*_ did not. Subjects hardly changed *L*_*lower*_ under environmental comfort conditions between March and October. This indicates that each of the *T*_*chest*_, *M*_*total*_, and *L*_*upper*_ was a factor in predicting *I*_*cl*_. *T*_*innermost*_ might also be a more influential factor than the clothing microclimate temperature.

## Introduction

Humans maintain heat balance through a heat exchange that occurs between their bodies and the surrounding thermal environment. This mechanism maintains thermal balance between conduction, convection, radiation, evaporation and heat production. The essential six factors that affect thermal comfort are air temperature, wind speed, radiation, humidity, activity, and clothing. Indeed, clothing plays the ultimate role in effectively protecting the human body by controlling the heat transfer from the body to the thermal environment or *vice versa*.

Gagge *et al*. [1] created the concept of the ‘clo’ unit to measure the thermal resistance of clothing, which was called ‘clothing insulation’. Clothing insulation as measured in clo indicates the characteristics of the heat transfer not of fabrics but of clothing to wear, and also takes into account the effect of air movement inside clothing through body movement or wind. Therefore, clothing insulation can be used to evaluate the effectiveness of clothing. Clothing insulation can be measured with human subjects or with a thermal manikin; however, both methods require the same laboratory conditions, including various types of apparatus specific facilities and other requirements, all of which are cost-intensive. Because of these restrictions, there has been considerable interest in developing different methods to predict the effects of clothing insulation [2,3].

Clothing microclimate temperature has also been used to evaluate the effects of clothing. Clothing microclimate generally refers to the air layer nearest to the skin when people wear clothing. Although the six factors stated above, including the value of clothing insulation (in clo), should be considered, the measurement of clothing microclimate temperature can offer a simple way to determine whether people feel comfortable in the clothing that they are wearing. Choi [4] explained that the capability of clothing in maintaining a climate, which could have an influence on human health, is worth further investigation. Since then, many studies on clothing microclimate temperature have been conducted, and various comfortable temperatures have been suggested [5]. Moreover, other studies have investigated relationships with other influential factors [6-11]. However, these studies have shown inconsistent relationships for clothing microclimate, and the results were not easy to compare with those of previous studies, because of differences between the studies in factors such as the types of environmental conditions or activities, and the variety of clothing used.

Nevertheless, clothing microclimate temperature has been used as a factor to assess the effects of clothing [12-15], and consequently, measurements of clothing microclimate have been used to develop protective outfits or investigate environmental conditions to which people are exposed. Some studies have been conducted to develop formulas for the estimation of clothing microclimate temperature [16,17] but those studies have dealt primarily with mean clothing microclimate temperature, which refers to measurements taken at several sites on the body. In addition, the formulas that have been developed are applicable only to specific conditions, thereby creating a gap in the literature for studies on the effects on comfort of clothing insulation and microclimate temperature under ordinary environmental conditions over the course of a year. Moreover, clothing and environmental designers might benefit from the ability to estimate clothing insulation from clothing microclimate temperature in terms of the development of functional clothing and designs for the workplace. People must feel comfortable in their working environment in order to maximize their productivity and efficiency at their work. The aim of this study was to investigate the relationship between factors related to clothing and clothing insulation under environmental temperatures within the comfort range over the course of a year in situations where people were allowed to select their own clothing to achieve their thermal comfort.

## Methods

### Subjects

In total, 20 people, (13 women, 7 men) took part in the study, which had the duration of 1 year (Table 1). All subjects were ordinary healthy students, and they were notified of the experimental procedures and the aim of the experiment.

**Table 1:** **Physical characteristics of subjects**^**a**^

**Sex**	**Age, years**	**Height, cm**	**Mass, kg**	**BSA, m**^**2**^
Male (n = 7)	24 (1.6)	174 (4.0)	71.9 (9.9)	1.86 (0.1)
Female (n = 13)	24 (2.5)	163 (5.8)	53.0 (8.5)	1.55 (0.1)

Abbreviations: *BSA*, Body surface area.

^a^Data are mean (SD).

### Environmental design

According to the study design, subjects were able to freely choose and wear their own clothing, given that the clothing selected should afford them thermal comfort under each environmental condition. Subjects were instructed to visit the climate chamber facility (that is, the facility in which the environmental temperatures were controlled for this experiment) several days before they took part in the experiments, in order to inquire about the conditions of the upcoming experiment, to allow them to decide how much and what type of clothing they would choose to wear for the actual experiments.

Each subject was repeatedly exposed to each *t*_*a*_ twice in a climate chamber between December 2002 and December 2003. The maximum atmospheric temperatures for the previous 5 years, as measured by the Meteorological Office were consulted, and the mean air temperature (*t*_*a*_) values for 12 months was determined. Six temperatures were chosen: 5°C (to represent January/February), 14°C (March/April), 25°C (May/June), 29°C (July/August), 23°C (September/October), and 8°C (November/December). The wind speed (0.14 ± 0.01ms^-1^) and humidity (45 ± 5%) in the chamber were held constant. Each experimental condition was determined based on the typical atmospheric temperatures, and the experiments were conducted each month for a year. This allowed subjects to choose their clothing based on its suitability for different conditions that were typical of several periods of the year.

### Measurements

A wet-bulb thermometer was used to measure *t*_*a*_ and air humidity. A kata thermometer was used to measure air velocity, and a black globe temperature was also measured every 10 minutes at the level of 0.6 m. The participants had their rectal temperature and their skin temperature (at seven body sites) measured every 5 minutes using K923 thermistors (Takara Instrument Co. Ltd., Yokohama Japan) and an equation from DuBois and DuBois [18] was used to measure mean skin temperature. Clothing microclimate temperatures were also measured at both the centre of the chest and in the interscapular area (*T*_*chest*_ and *T*_*scapular*_). The clothing temperature of the innermost layer (*T*_*innermost*_) was measured on this layer at 30 mm above the centre of the left breast every 5 minutes using a thermistor, which was fastened to the clothing with medical tape (Transpore; 3M Healthcare, St Paul, MN, USA). Metabolic rate was measured using a gas analyzer (Quark b^2^; COSMED Co., Rome, Italy) for 60 minutes. Body mass was measured before and after the 60 minute exposure period, using a digital multi-scale with an accuracy of 1 g (F150S, Sartorius Corp., Goettingen, Germany), and the resulting measurements were used to estimate evaporative loss over an hour. The physiological parameters measured were used to determine the value of the clothing insulation. Clothing insulation (*I*_*cl*_, measured in clo) was calculated with the formula specified in a previous study [19].

(1) $$I_{\mathrm{cl}}=\frac{5.55\left(T_{s}-t_{a}\right)A}{M-0.58P+0.83w\left(\frac{2\Delta T_{R}+\Delta T_{S}}{3}\right)}-I_{a}$$

(2) $$I_{a}=\frac{1}{0.61{\left(\frac{t_{a}}{298}\right)}^{3}+0.91\sqrt{V}\frac{298}{t_{a}}}$$

where *I*_*cl*_ is the overall insulation of assembly in clo units (clo); *I*_*a*_ is the insulation of air in clo; *T*_*s*_ is the skin temperature, excluding temperature of hands, head, and feet (°C), *t*_*a*_ is the ambient air temperature (°C); A is the body surface area [18] (m^2^); M is the total metabolic rate determined from oxygen consumption (kcal/hour); P is the evaporation loss estimated from successive weighings of clothed subjects (g/hour); w is the weight of the unclothed subject (kg); 0.83 is the composite specific heat of the human body (kcal/kg/°C); ∆T_R_ is the rate of fall of rectal temperature (°C/hour); ∆T_S_ is the rate of fall of mean skin temperature (°C/hour); and V is the air velocity (cm/s).

### Experimental procedure

Each subject was exposed to each experimental condition in the climatic chamber while wearing their own clothing that they considered gave them thermal comfort. Each participant was weighed while wearing only underwear and a disposable polypropylene gown (designated as ‘semi-nude’), and thermistors were then fitted on the participant to record skin, rectal, and clothing microclimate (*T*_*chest*_ and *T*_*scapular*_) temperatures. Subjects then put on their own clothing, and sat on a wooden chair for an hour. Subjects were allowed to listen to music or read books while their physiological responses and subjective responses, as well as the environmental conditions, were measured over the 60 minute exposure time. The subjects then removed the clothing and the thermistors at the end of the experiment, and were again weighed while semi-nude.

### Data analysis

Each subject was exposed to six different environmental conditions on two separate occasions, and participated in experiments 12 times over the period of a year (that is, 5°C (to represent January/February), 14°C (March/April), 25°C (May/June), 29°C (July/August), 23°C (September/October), and 8°C (November/December)). It has been previously suggested [2] that the range of *t*_*a*_ for thermal comfort can be approximately 15 to 28°C, thus the current study considered the environmental temperatures within this comfort range (that is, 14, 23, 25 and 29°C) for both the correlation and the regression models. The total number of trials for equation 1 was 126. The differences between clothing microclimate temperatures (*T*_*chest*_ and *T*_*scapular*_) and other parameters of environmental conditions were analyzed using a *t*-test. A planned comparison was made using a one-way analysis of variance (ANOVA) and a Pearson correlation analysis. *Post hoc* least significant difference tests were also carried out. A multivariate linear regression model was performed using a stepwise method. The unit of clothing mass used was the kilogram except in descriptive statistics for the sake of convenience.

## Results

### Differences in clothing-related factors between different environmental conditions

**Figure 1:**
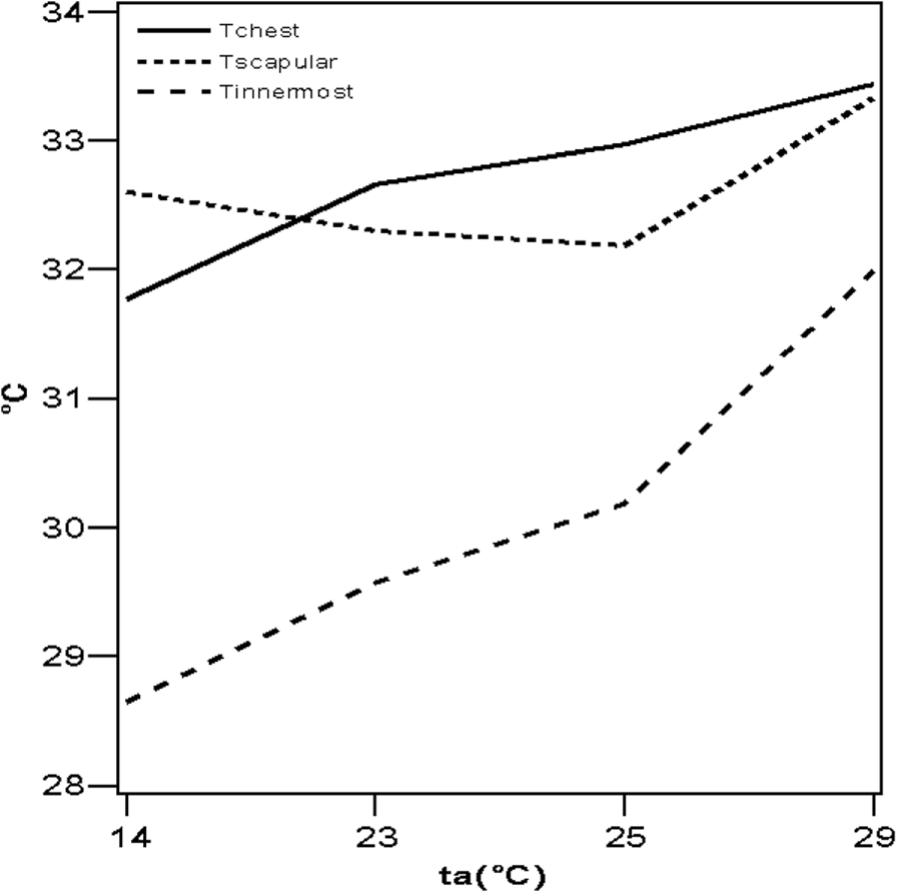
**Mean values of temperatures measured on the chest** (***T***_***chest***_**), between the shoulder blades (*T***_***scapular***_**), and on the innermost layer of clothing (*T***_***innermost***_**).**

Clothing microclimate temperatures were measured on the chest and in the interscapular area (*T*_*chest*_ and *T*_*scapular*_). Significant differences were seen in the clothing microclimate temperatures of the chest (*T*_*chest*_) compared with those of the interscapular areas (*T*_*scapular*_) (*P*<0.05). In addition, both *T*_*chest*_ and *T*_*scapular*_ were significantly different in the four different environmental conditions within the comfort range, and the differences between the lowest and the highest microclimate temperatures for the chest and the interscapular region (*T*_*chest*_ and *T*_*scapular*_) were 1.9 and 1.2°C, respectively. *T*_*chest*_ at 14°C was significantly different from *T*_*chest*_ at 25 and 29°C, but *T*_*chest*_ at 23°C was not different from *T*_*chest*_ at the other three air temperature (*t*_*a*_) values. *T*_*scapular*_ showed a slight difference at 29°C compared with the other three air temperature (*t*_*a*_) values. The clothing temperature of the innermost layer (*T*_*innermost*_) showed differences between each air temperature (*t*_*a*_), and the difference between the lowest and the highest temperature was 3.5°C (*P*<0.05) (Table 2, Figure 1). *T*_*innermost*_ at 14 and 29°C was different from the reading at the three other temperatures; however, there was no difference in *T*_*innermost*_ at 23 or 25°C.

**Table 2:** **The results of ANOVA analysis and clothing microclimate temperature after 60 minutes of exposure**^**a**^

***t***_***a,***_**°C (months)**	***T***_***chest,***_**°C**	***T***_***scapular,***_**°C**	***T***_***innermost,***_**°C**
14 (Mar/Apr)	31.6(3.00)^b,c^	32.3(2.14)^b^	28.6(2.17)^b,c,d^
23 (Sept/Oct)	32.4(2.01)	32.2(1.54)^c^	29.6(1.31)^b,d^
25 (May/Jun)	33.1(1.78)^b^	32.2(1.30)^d^	30.2(1.36)^c,e^
29 (Jul/Aug)	33.5(1.45)^c^	33.4(0.86)^b,c,d^	32.0(1.02)^d,d,e^
*F*-value	4.355	3.901	22.269
*P*	0.006	0.011	0.000

Abbreviations: *T*_*chest*_, Temperature on the chest; *T*_*innermost*_, Temperature of the innermost layer of clothing; *T*_*scapular*_, Temperature in the interscapular area.

^a^Data are mean (SD).

^b–d^Superscript letters indicate *post hoc* tests; identical letters mean the differences between air temperatures within each parameter.

The level of clothing insulation (*I*_*cl*_) varied significantly between the different air temperature (*t*_*a*_) values when subjects were seated at rest while wearing their own clothing. The difference between the highest and the lowest clothing insulation (*I*_*cl*_) was 1.32 clo under environmental conditions within the comfort range. The results of *I*_*cl*_ at 23°C aligned only with those of *I*_*cl*_ at 25°C, whereas the *I*_*cl*_ at 14 and 29 °C differed from that at the other air temperature (*t*_*a*_) values. The *post hoc* analysis of *I*_*cl*_ showed the same results as that of *T*_*innermost*_. Total clothing mass (*M*_*total*_) and the total number of layers within an outfit (*L*_*total*_) also showed differences between each air temperature (*t*_*a*_) value. The difference between the highest and the lowest clothing mass (*M*_*total*_) was 1186 g, and the difference between the highest and the lowest numbers of total layers was 2.1 layers. The figures for *M*_*total*_ differed at all tested air temperature (*t*_*a*_) values. However, *L*_*total*_ at 14 and 23 °C showed a difference from that at the other air temperature (*t*_*a*_) values, and *L*_*total*_ at 25°C showed no difference from that at 29°C. The number of upper clothing layers (*L*_*upper*_) showed a similar tendency to differences as that of the total layers (*L*_*total*_) within an outfit, while the *post hoc* results for *L*_*upper*_ were the same as those for *L*_*total*_. However, the number of lower clothing layers (*L*_*lower*_) showed no difference between all air temperature (*t*_*a*_) values (Table 3, *P*<0.05).

**Table 3:** **The results of ANOVA analysis between air temperature and various parameters**^**a**^

***t***_***a***_**, °C**	**Number of layers of clothing within an outfit**			***M***_***total***_**, g**	***I***_***cl***_**, clo**
	**Upper clothing**	**Lower clothing**	**Total**		
14 (Mar/Apr)	3.2(1.01)^b,c,d^	1.0(0.00)	4.2(1.01)^b,c,d^	1659(370.3)^b,c,d^	2.10(0.362)^b,c,d^
23 (Sept/Oct)	1.8(0.64)^b,e,f^	1.0(0.00)	2.8(0.64)^b,e,f^	928(248.0)^b,e,f^	1.37(0.392)^b,e^
25 (May/Jun)	1.4(0.49)^c,e^	1.0(0.18)	2.4(0.56)^c,e^	732(225.5)^c,e,g^	1.38(0.402)^c,f^
29 (Jul/Aug)	1.1(0.35)^d,f^	1.0(0.00)	2.1(0.35)^d,f^	473(207.5)^d,f,g^	0.78(0.241)^d,e,f^
*F*-value	59.924	1.046	56.841	109.727	71.361
*P*	0.000	0.375	0.000	0.000	0.000

Abbreviations: *I*_*cl*_, Level of clothing insulation; *M*_*total*_, Total clothing mass.

^a^Data are mean (SD).

^b–g^Superscript letters indicate *post hoc* tests; identical letters mean the differences between *t*_*a*_ values within each parameter.

### Relationships between mass, layer and microclimate of clothing and clothing insulation

*T*_*chest*_ had significant positive relationships with *T*_*scapular*_, *T*_*innermost*_, and air temperature (*t*_*a*_). The clothing microclimate temperatures (*T*_*chest*_ and *T*_*scapular*_) and *T*_*innermost*_ increased as *t*_*a*_ went up (Table 2, Table 3, Table 4). However, *T*_*chest*_ showed significant inverse relationships with *I*_*cl*_ and *M*_*total*_, but no relationships with *L*_*upper*_, *L*_*lower*_, or *L*_*total*_. *T*_*chest*_ increased as *I*_*cl*_ and *M*_*total*_ both decreased. *T*_*scapular*_ showed significant positive relationships with *T*_*innermost*_ and *t*_*a*_, and *T*_*scapular*_ increased as *T*_*innermost*_ and *t*_*a*_ increased. By contrast, no relationship was seen between *T*_*scapular*_ and other parameters (Table 4).

**Table 4:** Correlation coefficients for the various parameters

**Clothing-related parameters**	***I***_***cl***_	***T***_***chest***_	***T***_***scapular***_	***L***_***upper***_	***L***_***lower***_	***L***_***total***_	***M***_***total***_	***T***_***innermost***_
*I*_*cl*_	1	−0.217^a^	–	0.617	–	0.612	0.629	−0.413
*T*_*chest*_	−0.217^a^	1	0.228^a^	–	–	–	−0.328	0.370
*T*_*scapular*_	–	0.228^a^	1	–	–	–	–	0.207^a^
*L*_*upper*_	0.617	–	–	1	–	0.996	0.723	−0.306
*L*_*lower*_	–	–	–	–	1	–	–	–
*L*_*total*_	0.612	–	–	0.996	–	1	0.717	−0.306
*M*_*total*_	0.629	−0.328	–	0.723	–	0.717	1	−0.535
*T*_*innermost*_	−0.413	0.370	0.207^a^	−0.306	–	−0.306	−0.535	1
*t*_*a*_	−0.783	0.305	0.179^a^	−0.765	–	−0.758	−0.849	0.578

Abbreviations: *clo*, Clothing units; *I*_*cl*_, Level of clothing insulation; *L*_*lower*_, The number of lower clothing layers; *L*_*total*_, The number of total layers within an outfit; *L*_*upper*_, The number of upper clothing layers; *M*_*total*_, Total clothing mass; *t*_*a*_, Air temperature; *T*_*chest*_, Temperature on the chest; *T*_*innermost*_, Temperature on the innermost layer of clothing; *T*_*scapular*_, Temperature in the interscapular area.

^a^Significant at *P* < 0.05; all other values were significant at *P* <0.01.

Cells with ‘–’mean that no relationship occurred.

The relationship of both *T*_*innermost*_ and *T*_*chest*_ with other factors showed a similar tendency (Table 4). *T*_*innermost*_ had a significant positive relationship with *t*_*a*_, and *T*_*innermost*_ increased as *t*_*a*_ increased. However, *T*_*innermost*_ had a significant inverse relationship with *I*_*cl*_, *M*_*total*_, *L*_*upper*_, and *L*_*total*_; *T*_*innermost*_ increased as *I*_*cl*_, *M*_*total*_, and the number of clothing layers (*L*_*upper*_ and *L*_*total*_) decreased. *M*_*total*_ showed positive relationships with both *L*_*upper*_ and *L*_*total*_ and *L*_*upper*_ had a positive relationship with *L*_*total*_. *M*_*total*_ increased as the number of clothing layers (*L*_*upper*_ and *L*_*total*_). increased. However, *L*_*lower*_ did not show any relationship with any of the other parameters measured (Table 4). Therefore, *L*_*upper*_ had an influence on the other parameters but *L*_*lower*_ did not. Additionally, *I*_*cl*_, *M*_*total*_, *L*_*upper*_, and *L*_*total*_ had inverse correlations with *t*_*a*_.

At the beginning of a stepwise regression analysis, *t*_*a*_ was selected out of all the parameters measured (that is, *t*_*a*_, *T*_*chest*_, *T*_*scapular*_, *T*_*innermost*_, *M*_*total*_, *L*_*upper*_, *L*_*lower*_, and *L*_*total*_) and the regression function using *t*_*a*_ could be derived with 63% of variance for the estimation of *I*_*cl*_. However, the aim of this study was to identify the relationship between clothing insulation (*I*_*cl*_) and clothing-related parameters, and a stepwise regression analysis was conducted with all parameters except *t*_*a*_. A multivariate linear regression model was then derived (3), and clothing insulation (*I*_*cl*_) could be estimated using total clothing mass (*M*_*total*_), clothing microclimate temperature in the interscapular region (*T*_*scapular*_) and the number of upper clothing layers (*L*_*upper*_) under comfortable environmental conditions (Table 5). The standardized coefficient value was 0.387 of total clothing mass (*M*_*total*_), and 0.380 for upper clothing layers (*L*_*upper*_). The total clothing mass (*M*_*total*_) and the number of upper clothing layers (*L*_*upper*_) had a similar influence on clothing insulation (*I*_*cl*_), and the clothing microclimate temperature in the interscapular region (*T*_*scapular*_) had less influence on *I*_*cl*_ than the total clothing mass (*M*_*total*_) and the number of upper clothing layers (*L*_*upper*_) (Table 5).

**Table 5:** **Regression coefficients for each explanatory variable of clothing insulation (*I***_***cl***_**)**^**a,b**^

**Explanatory variable**	**B**	**SE**	**β**	***P*value**
Constant	3.552	0.872	–	0.000
*M*_*total*_, kg	0.424	0.110	0.387	0.000
*L*_*upper*_, n	0.202	0.054	0.380	0.000
*T*_*scapular*_, °C	−0.089	0.027	−0.227	0.001

Abbreviations: *L*_*upper*_, The number of upper clothing layers; *M*_*total*_, Total clothing mass; *T*_*scapular*_, Temperature in the interscapular area.

^a^r^2^ = 0.535, adjusted r^2^ =0 .521.

^b^*I*_*cl*_ = 3.552 + 0.424*M*_*total*_ + 0.202*L*_*upper*_ − 0.089*T*_*scapular*_; r = 0.731 (3).

Provided that total clothing mass (*M*_*total*_), the number of upper clothing layers (*L*_*upper*_) , and clothing microclimate temperature in the scapular area (*T*_*scapular*_) are known, then under comfortable environmental conditions (air temperatures between 14 and 29°C), clothing insulation can be predicted by equation (3). For example, if *M*_*total*_, *L*_*upper*_, and *T*_*scapular*_ are 0.75 kg, 2 layers, and 31 °C respectively, clothing insulation (*I*_*cl*_) would be 1.52 clo. If *M*_*total*_, *L*_*upper*_, and *T*_*scapular*_ are 0.5 kg, 1 layer, and 33 °C respectively, clothing insulation (*I*_*cl*_) would be 1.03 clo.

## Discussion

All clothing factors measured including clothing microclimate temperatures (*T*_*chest*_ and *T*_*scapular*_) and the clothing temperature of the innermost layer (*T*_*innermost*_) showed differences between the four different air temperatures (*t*_*a*_) values (14, 23, 25, and 29 under environmental conditions within the range of comfort) while subjects wore the clothing they considered necessary for comfort between March and October. Clothing microclimate temperatures on the chest and in the interscapular area (*T*_*chest*_ and *T*_*scapular*_) differed considerably between the four different air temperature (*t*_*a*_) values and there was the difference between the clothing microclimate (*T*_*chest*_ and *T*_*scapular*_) Clothing insulation (*I*_*cl*_) showed significant inverse correlations with clothing microclimate temperature on the chest (*T*_*chest*_) and air temperature (*t*_*a*_) values of 14, 23, 25, and 29°C, and clothing microclimate temperature (*T*_*chest*_ and *T*_*scapular*_) was highest in summer. Seol *et al*. [20] also found that the clothing microclimate temperature was highest during summer and lowest during winter. They investigated clothing microclimate temperature by region, season, gender and age and microclimate temperature on the chest was measured. The range of microclimate temperature over four seasons was 29.7-33.3 °C. Kwon and Choi [3] noted that air temperature (*t*_*a*_) was one of the essential factors affecting clothing insulation. In the current study, it was shown that air temperature (*t*_*a*_) influences even the microclimate temperature on the chest (*T*_*chest*_) and the clothing temperature of the innermost layer (*T*_*innermost*_) under comfortable environmental conditions. The two clothing microclimate temperatures had positive relationships with the clothing temperature of the innermost layer (*T*_*innermost*_) and the latter fell between the clothing microclimate temperatures and the air temperature (*t*_*a*_) (Table 2). Park and Choi [17] also reported that the environmental temperature was lower than the clothing microclimate temperature and the microclimate temperature was lower than the skin temperature, and they described this using the term ‘temperature gradient’. The clothing temperature of the innermost layer might be a useful predictor of clothing microclimate temperatures. In addition, a *post hoc* analysis of the clothing temperature of the innermost layer (*T*_*innermost*_) gave the same results as the analysis of clothing insulation (*I*_*cl*_), and thus it is likely that the clothing temperature would be a helpful predictor of the appropriate clothing insulation. If the relationship between clothing insulation and microclimate temperature is investigated in a particular environment, it could conflict with the result of the current study. For example, the previous study [17] showed that microclimate temperature rose as the amount of clothing insulation increased at a single environmental temperature. It was conducted by controlling clothing factors under specific environmental conditions, and might be useful to apply to the effect of clothing on health. However, those kinds of relationships would not occur when people are exposed to actual environments because people have lower skin temperatures in the winter than they do in the summer, and there are large difference in environmental conditions between the seasons such as between summer and winter. Furthermore, the microclimate temperature on the chest seems to be influenced by clothing choice, such as the type of neckline worn and clothing behavior, such as buttoning clothing or leaving it open, as a previous study [21] found that the position of clothing openings had an influence on the clothing microclimate.

In the current study, we found that the clothing temperature of the innermost layer (*T*_*innermost*_) showed higher correlations with other parameters than did the microclimate temperature on the chest (*T*_*chest*_). The number of clothing layers (*L*_*upper*_*, L*_*lower*_ and *L*_*total*_) did not correlate with clothing microclimate temperatures (*T*_*chest*_ and *T*_*scapular*_), but the total clothing mass (*M*_*total*_) did show a relationship with clothing microclimate temperature on the chest (*T*_*chest*_). Although the numbers of clothing layers (*L*_*total*_ and *L*_*upper*_) decreased as air temperature increased, the difference in the numbers of clothing layers at 23, 25, and 29°C was about one layer. Therefore, the numbers of clothing layers seemed to have no relationship with the microclimate temperatures (*T*_*chest*_ and *T*_*scapular*_).

In a study using a thermal manikin, Choi [22] found that a strong relationship existed between clothing insulation and clothing microclimate temperature. In general, studies that have used a thermal manikin have been conducted while maintaining a certain surface temperature, and such studies gave a higher correlation coefficient than the current study, which was conducted with human subjects during the course of 1 year. The clothing microclimate depends on the layers of air between the clothing and the skin, and the clothing microclimate temperature is generally measured at the chest and interscapular area. A number of studies have suggested that clothing microclimate temperature on the chest is significantly related to physiological factors and clothing-related characteristics [5-7,23-26]. Hence, the use of clothing microclimate temperatures to predict clothing insulation under various environmental conditions may be convenient.

In the present study, we found a significant correlation for clothing microclimate temperature on the chest (*T*_*chest*_) and the clothing insulation (*I*_*cl*_), but the correlation coefficient was low. Seasonal effects might also influence this low correlation coefficient. Several studies have suggested that the temperatures at which people feel comfortable in the summer are higher than the temperatures at which they feel comfortable in the winter. Such findings have been supported by other studies on clothing microclimate temperature and skin temperatures [7,27,28]. Earlier studies also indicated that people had a tendency to choose and wear particular clothing based not on environmental temperature but rather on season [3,29]. In addition, subjects in the current study varied in their individual clothing behavior (for example, wearing a neckline buttoned up or partially closed because the way of wearing ensemble was not controlled in other words, participants felt free to put on clothing. Furthermore, air layers may have been affected by the convection caused by even small body movements, even though the subjects were at rest. Clothing microclimate temperature on the chest (*T*_*chest*_) at 23°C did not show any difference from that at other air temperatures. Therefore, there does not seem to be a relationship between the number of clothing layers and clothing microclimate temperatures, even though Chu [30] also noted that the rate of ventilation was affected by the opening area of clothing.

To accurately evaluate the effects of clothing, the clothing insulation, physical characteristics of the thermal environment, and physiological responses should be measured while people are wearing the clothing. However, if necessary (for example, if there is a lack of equipment or difficulties with experimental procedures), simpler measurements, such as clothing microclimate temperature, clothing weight, and the number of clothing layers could be used. The formula we provide here can be applied to environments that are analogous to those in the current study because it has been derived using an empirical method. However, clothing microclimate is influenced by a variety of factors, such as the characteristics of the fabric, types of openings, fit of the clothing, and environmental and physiological factors. Thus, the clothing temperature of the innermost layer might be another factor to predict the effect of clothing and clothing insulation. Further studies on the clothing temperatures of the innermost layer should be conducted in hot and cold environmental conditions. It may also be meaningful to locate the sites that give the most representational temperature of the innermost clothing layer.

## Conclusion

In this study, each subject was exposed to each different experimental condition in the climatic chamber over the course of a year while wearing their own clothing for thermal comfort. The differences and relationships between air temperature and other factors related to the clothing were investigated, and we obtained the following findings. 1) All clothing factors measured, except the number of lower clothing layers (*L*_*lower*_), showed differences between environmental conditions (that is, 14, 23, 25, and 29 °C; *P*<0.05). The ranges of average clothing microclimate temperatures were 31.6 to 33.5°C on the chest (*T*_*chest*_) and 32.2 to 33.4 °C in the interscapular area (*T*_*scapular*_). The range of average clothing temperature of the innermost layer (*T*_*innermost*_) was 28.6 to 32.0 °C. The average number of upper clothing layers (*L*_*upper*_) ranged from 1.1 to 3.2 layers and that of the total clothing mass (*M*_*total*_) ranged from 473 to 1659 g respectively. The range of clothing insulation (*I*_*cl*_) was 0.78 to 2.10 clo. 2) The *post hoc* analyses gave inconsistent results for most of the clothing-related parameters measured. However, the analysis of *T*_*innermost*_ produced the same results as that of *I*_*cl*_. Likewise, the analysis of the number of upper clothing layers (*L*_*upper*_) gave the same result as that of the number of total layers (*L*_*total*_) within an outfit. 3) The air temperature (*t*_*a*_) showed positive relationships with the clothing microclimate temperatures (*T*_*chest*_ and *T*_*scapular*_) and with *T*_*innermost*_, but inverse correlations with *I*_*cl*_, *M*_*total*_, and the numbers of clothing layers (*L*_*upper*_ and *L*_*total*_) *T*_*chest*_, *T*_*scapular*_ and *T*_*innermost*_ increased as *t*_*a*_ went up. 4) *I*_*cl*_ had inverse relationships with *T*_*chest*_ and *T*_*innermost*_, but positive relationships with *M*_*total*_ and the numbers of clothing layers (*L*_*upper*_ and *L*_*total*_). Clothing insulation (*I*_*cl*_) could be estimated using total clothing mass (*M*_*total*_), the number of upper clothing layers (*L*_*upper*_), and the clothing microclimate temperature in the interscapular area (*T*_*scapular*_), using a multivariate linear regression model. 5) *L*_*upper*_ had positive relationships with *I*_*cl*_ and *M*_*total,*_ but *L*_*lower*_ did not. Subjects hardly changed the lower layers (*L*_*lower*_) they wore under comfortable environmental conditions between March and October.

These results indicate that each of the clothing microclimate temperature on the chest, the total clothing mass, and the number of upper clothing layers was a factor in predicting clothing insulation, and that the clothing temperature of the innermost layer could be a more influential factor than the clothing microclimate temperature or other clothing-related factors on clothing insulation under environmental conditions within the comfort range.

## Competing interests

The authors declare that they have no competing interests.

## Authors’ contributions

JC supervised the study and designed the experiments. JK performed the experiments, analyzed the data and wrote the manuscript. JC and JK interpreted the data and JK edited the manuscript with advice of JC. Both authors read and approved the final manuscript.
